# Supplementary material for: Tumor-derived stearic acid induces macrophage Egr2 signaling to suppress anti-tumor immunity in breast cancer
Source: bioRxiv. 2026 Jul 21:2026.07.15.738781. Preprint. [Version 1] doi: 10.64898/2026.07.15.738781 (PMC13419751; doi:10.64898/2026.07.15.738781)
Supplement: Supplement 1 [file media-1.pdf]

Online Supplemental Data

Tumor-derived stearic acid induces macrophage Egr2 signaling to suppress anti-tumor immunity in breast cancer

Yongling Ning[2], Caijun Wu[2], Sailesh Phuyal[2], Xiaoling Hu[2], Hong Li[2], Robert A Mitchell[1,2], Jun Yan[1, 2, *], Chuanlin Ding[1, 2, *]

[1] The Hiram C. Polk, Jr., MD Department of Surgery, Division of Immunotherapy, UofL Health Brown Cancer Center, University of Louisville School of Medicine, Louisville, KY, USA

[2] UofL Health Brown Cancer Center, University of Louisville School of Medicine, Louisville, KY, USA

Supplemental Table S1. Antibodies used in Flow cytometry and Western blotting

**Antibodies**	**Company and catalog number**
PerCP/Cyanine5.5 anti-mouse CD45	BioLegend, No. 157208
PE/Cyanine7 anti-mouse CD45	BioLegend, No. 103114
APC anti-mouse/human CD11b	BioLegend, No. 101212
PE anti-mouse Ly6G	BioLegend, No. 127608
PerCP anti-mouse Ly6C	BioLegend, No. 128028
PE anti-mouse F4/80	BioLegend, No. 123110
APC anti-mouse CD4	BioLegend, No. 100412
FITC anti-mouse CD8a	BioLegend, No. 100706
APC anti-mouse NK-1.1	BioLegend, No. 108710
PE-anti-mouse TNFα	BioLegend, No. 506306
PE anti-mouse IFN-γ	BioLegend, No. 505808
PE-Cy7 anti-mouse IFN-γ	BioLegend, No. 505826
FITC anti-mouse I-A/I-E	BioLegend, No. 107606
APC anti-mouse Egr2	Invitrogen, No. 17-6691-82
Fixable Viability Dye eFluor™ 780	Thermo Fisher Scientific, No. 65-0865-14
Anti-mouse PPARγ	Cell Signaling, No. 2443

Supplemental Table S2. Antibodies used in CyTOF and IMC

	**Antibodies**	**Company and catalog number**
1	Anti-Mouse CD45 (30-F11)-89Y	Fluidigm, No. 3089005B
2	Anti-Mouse CD11c (N418)-142Nd	Fluidigm, No. 3142003B
3	Anti-Mouse CD69 (H1.2F3)-143Nd	Fluidigm, No. 3143004B
4	Anti-Mouse CD4 (RM4-5)-145Nd	Fluidigm, No. 3145002B
5	Anti-Mouse F4/80 (BM8)-146Nd	Fluidigm, No. 3146008B
6	Anti-Mouse CD103 (2E7)-148Nd	Biolegend, No. 121402
7	Anti-Mouse CD19 (6D5)-149Sm	Fluidigm, No. 3149002B
8	Anti-Mouse Ly-6C (HK1.4)-150Nd	Fluidigm, No. 3150010B
9	Anti-Mouse CD25 (3C7)-151Eu	Fluidigm, No. 3151007B
10	Anti-Mouse CD3e (145-2C11)-152Sm	Fluidigm, No. 3152004B
11	Anti-Mouse CD274/PD-L1-153Eu (10F.9G2)	Fluidigm, No. 3153016B
12	Anti-Mouse PD-1 (29F.1A12)-159Tb	Fluidigm, No. 3159024B
13	Anti-Mouse CD62L (MEL-14)-160Gd	Fluidigm, No. 3160008B
14	Anti-Human/Mouse CD44 (IM7)-162Dy	Fluidigm, No. 3162030B
15	Anti-Mouse CX3CR1 (SA011F11)-164Dy	Fluidigm, No. 3164023B
16	Anti-Mouse CD8a (53-6.7)-168Er	Fluidigm, No. 3168003B
17	Anti-Mouse CD206/MMR (C068C2)-169Tm	Fluidigm, No. 3169021B
18	Anti-Mouse NK1.1 (PK136)-170Er	Fluidigm, No. 3170002B
19	Anti-Mouse CD11b (M1/70)-172Yb	Fluidigm, No. 3172012B
20	Anti-Mouse CD223/LAG3 (C9B7W)-174Yb	Fluidigm, No. 3174019B
21	Anti-Human/Mouse CD45R/B220 (RA3-6B2)-176Yb	Fluidigm, No. 3176002B
22	Anti-Mouse I-A/I-E (M5/114.15.2)-209Bi	Fluidigm, No. 3209006B
23	Anti-Mouse CD127/IL7Ra (A7R34)-175Lu	Fluidigm, No. 3175006B
24	Anti-Mouse iNOS (CXNFT)-161Dy	Fluidigm, No. 3161011B
24	Anti-Mouse TNFα (MP6-XT22)-141Pr	Fluidigm, No. 3141013B
25	Anti-Mouse IL-2 (JES6-5H4)-144Nd	Fluidigm, No. 3144002B
26	Anti-Mouse CCR2 (475301R)-156Gd	R&D System, No. MAB55381R
27	Anti-Mouse IFNg (XMG1.2)-165Ho	Fluidigm, No. 3165003B
28	Anti-Mouse IL-6 (MP5-20F3)-167Er	Fluidigm, No. 3167003B
29	Anti-Mouse Foxp3 (FLK-16s)-158Gd	Fluidigm, No. 3158003A
30	Anti-Human CD8a (C8/144B)-162Dy	Standard BioTools, No. 3162034D
31	Anti-Human CD68 (KP1)-141Pr	Standard BioTools, No. 91H012141
32	Anti-Egr2	GeneTex, No. GTX102912

Supplemental Table S3. Primer sequences for real-time PCR

Gene	Forward primer	Reverse primer
CEBPB	ACTTCAGCCCCTACCTGGAG	GGCTCACGTAACCGTAGTCG
PPARG	TGTCGGTTTCAGAAGTGCCT	CCAACAGCTTCTCCTTCTCG
CHIL3	ACTTTGATGGCCTCAACCTG	AATGATTCCTGCTCCTGTGG
ARG1	TTTTAGGGTTACGGCCGGTG	CCTCGAGGCTGTCCTTTTGA
RETNLA	CTCATCTGCATCTCCCTGCT	AGGAGGCCCATCTGTTCATAG

Supplemental Figure S1

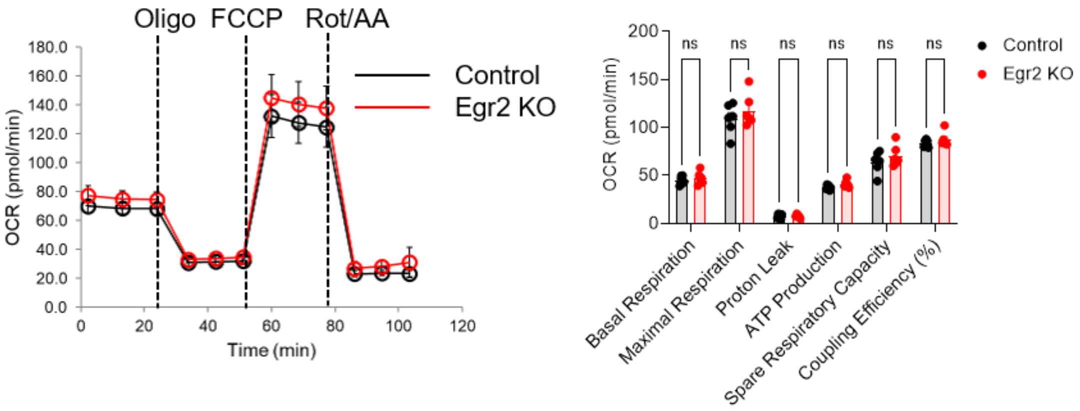

Figure S1. Seahorse Mito stress test in TAMs from E0771 tumors of control and Egr2 KO mice. Summarized of relative values of ECAR bioenergenic profiling was shown. ns: not significant.

Supplemental Figure S2

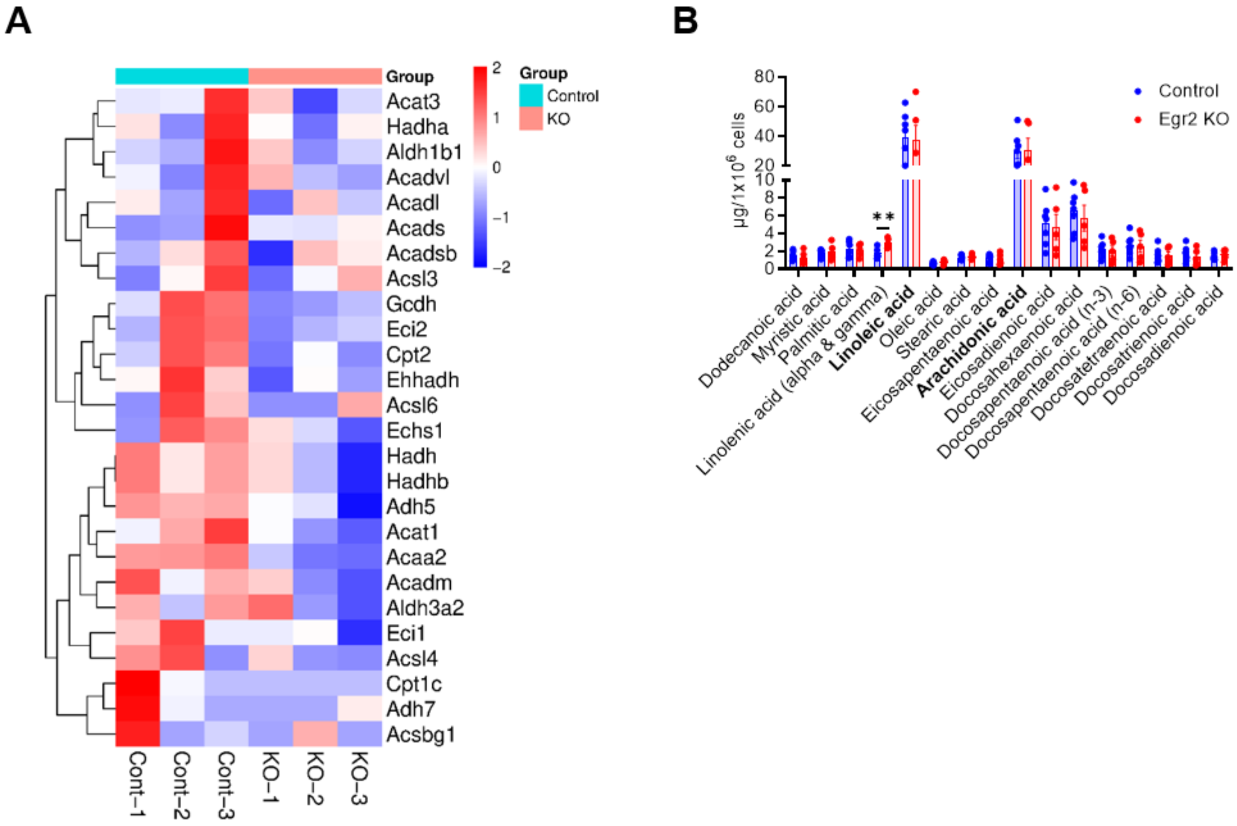

Figure S2. (A) Heatmap showing the clustering of fatty acid degradation related genes in TAMs from E0771 tumor-bearing control and Egr2 KO mice (n=3) based on log-relative abundances. (B) Contents and levels of fatty acid in the lysates of TAMs from E0771 tumor-bearing control and Egr2 KO mice.

Supplemental Figure S3

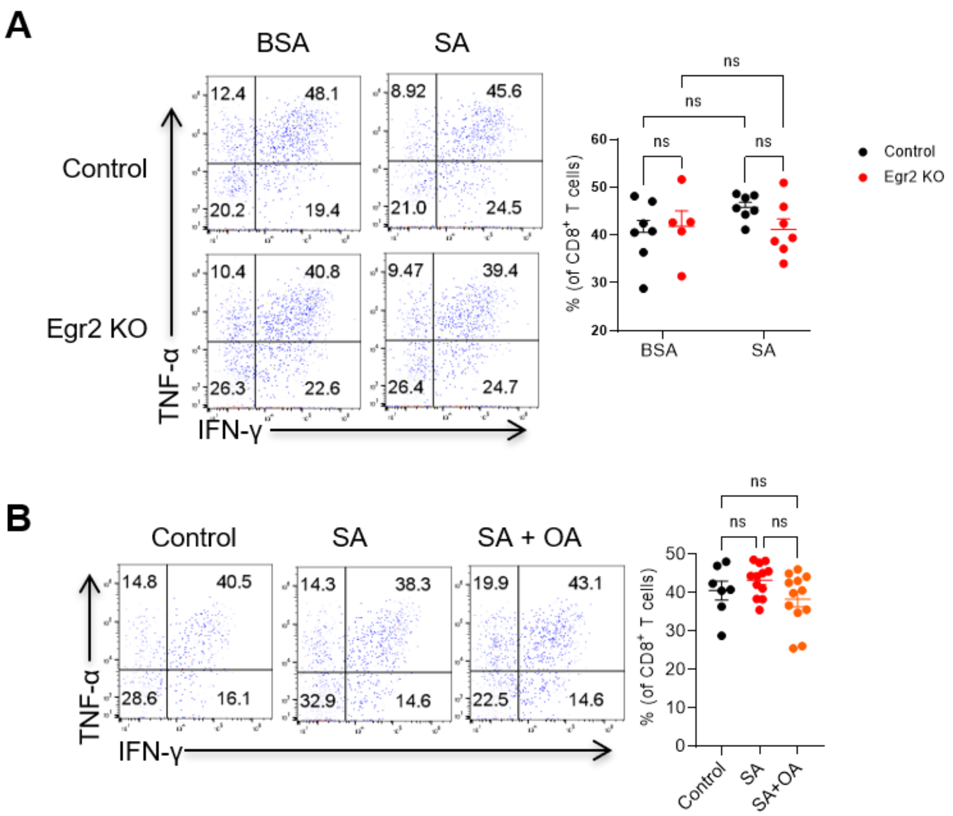

Figure S3. (A) Percentages of IFN-γ^+TNF-α^+ CD8$^+$ T cells on day 7 in tumor cell and macrophage admix experiments in 4 groups**.** (B) Percentages of IFN-γ^+TNF-α^+ CD8$^+$ T cells on day 7 in tumor cell and macrophage admix experiments in 3 groups. Each dot represents an individual mouse. ns: not significant.
